# Prevalence of Overweight Among Elementary and Middle School Students in Mississippi Compared With Prevalence Data From the Youth Risk Behavior Surveillance System

**Published:** 2006-06-15

**Authors:** Elaine Fontenot Molaison, Jerome R Kolbo, Mary Kay Meyer, Nancy M Speed, Alan D Penman, Lei Zhang

**Affiliations:** Department of Nutrition and Food Systems, The University of Southern Mississippi; The University of Southern Mississippi, Hattiesburg, Miss; The University of Southern Mississippi, Hattiesburg, Miss; The University of Southern Mississippi, Hattiesburg, Miss; The University of Mississippi Medical Center, Jackson, Miss; Mississippi Department of Health, Jackson, Miss

## Abstract

**Introduction:**

The purpose of the Child and Youth Prevalence of Overweight Survey was to estimate the prevalence of overweight and at risk for becoming overweight among children in Mississippi (grades 1–8) using height and weight measures instead of self-report and to compare the findings for grades 6 through 8 with data from the Youth Risk Behavior Surveillance System for middle school students (grades 6–8).

**Methods:**

Students in randomly selected classes from 37 sampled elementary and middle schools throughout Mississippi participated in the study. School staff were trained to collect height and weight data using a standardized procedure.

**Results:**

Overall, 24.0% of students in grades 1 through 8 were found to be overweight, and another 14.7% were at risk for becoming overweight. With the exception of sixth grade, there was a trend of increasing prevalence of overweight by grade (17.5% in grade 1 compared with 31.3% in grade 8). In the Child and Youth Prevalence of Overweight Survey, 25.2% of students in grades 6 through 8 were found to be overweight, compared with 18.5% in the Youth Risk Behavior Surveillance System.

**Conclusion:**

A high percentage of students in Mississippi are already overweight in first grade, and the prevalence tends to increase by grade. Data collected from middle school students through measured heights and weights in the Child and Youth Prevalence of Overweight Survey were higher than self-reported data from the Youth Risk Behavior Surveillance System. Our data suggest that self-reported data underestimate the prevalence of overweight among middle school students. Efforts to monitor students' body mass index and assess effectiveness of interventions should include all grades and use measured heights and weights rather than self-reports.

## Introduction

Since the 1970s, the prevalence of overweight among children and youth has increased dramatically. In the 1999–2000 National Health and Nutrition Examination Survey (NHANES), 15.3% of children aged 6 to 11 years were overweight, compared with 4.0% in the 1970–1974 NHANES and 11.3% in the 1988–1994 NHANES (1). The increase in prevalence of overweight has differed by race, sex, and geographic location. The greatest rates of increase have been among African American girls, Hispanic youth, and children living in the South, with approximately 4 in 10 African American and Hispanic children and youth being overweight or at risk for becoming overweight (1,2). The percentage of overweight children in the South increased from 9.8% in 1986 to 17.1% in 1998, whereas the increase in the rest of the country during the same period was from 7.6% to 10.8% (1,2).

Mississippi is one of the southern states that has experienced dramatic increases in overweight among children and youth. Of 32 states with weighted data from the 2003 Youth Risk Behavior Surveillance System (YRBSS) reported by the Centers for Disease Control and Prevention (CDC) (3), Mississippi had the highest percentage of high school students in the nation who were overweight (15.7%) and one of the highest percentages of students at risk for becoming overweight (15.7%). The reported rates were slightly higher than in 2001, when 14.0% were overweight and another 15.4% were at risk for becoming overweight (4).

Since 2001, Mississippi has also collected data through the YRBSS on public middle school students. In 2003, 18.5% of students in grades 6 through 8 were overweight and 20.7% were at risk for becoming overweight (5). As with the older students, these rates were higher than in 2001, when 16.2% of the middle school students were overweight, and another 18.3% were at risk for becoming overweight (6).

With an increased awareness of overweight among youth in recent years have come more established links between overweight and social and emotional (7-10), academic (11-14), and physical health problems (15-19) in youth and adults. Research suggests that the greatest risk for overweight children and youth is likely to be the persistence of elevated body mass into adulthood (16,20-23). Even though the likelihood for children and youth to become overweight or obese in adulthood increases with the age at which they become overweight (16), few studies have examined the prevalence rates of overweight or at risk for becoming overweight among younger school-aged children (24). In addition, although research using self-reported data has indicated that the rates continue to increase and vary by race, sex, and geographic location, few studies have established prevalence rates through the use of objective measurements of height and weight and differentiated these rates by age, sex, and race (25-27).

The purpose of this study was to collect anthropometric height and weight data on both elementary and middle school students (grades 1–8) and to compare these findings with comparable data collected through the YRBSS for middle school students (grades 6–8). This study assessed both overweight and at risk of overweight by grade, sex, and racial and ethnic status. As decisions are made for prevention and treatment programs, we hope that more precise prevalence data will clarify the nature and extent of the problem and assist decision makers in deciding where and at whom to focus their efforts.

## Methods

### Child and Youth Prevalence of Overweight Survey 

The Child and Youth Prevalence of Overweight Survey (CAYPOS) was conducted in April and May 2003 in Mississippi. Before beginning the study, institutional review board approval was received through the Human Subjects Protection Review Committee at The University of Southern Mississippi. The sampling frame consisted of all elementary and middle school grades 1 through 8 (N = 729) with fall 2002 enrollment numbers by grade. A sample of 57 schools was selected using PC Sample software (28), which selects schools by probability proportional to enrollment with a random start. After agreement to participate was obtained from school principals, a list of all classes meeting during the second period of the day was requested. Classes were then selected by a systematic equal probability sampling with a random start.

After schools agreed to participate and classes were selected, measuring equipment (i.e., scales, metal measuring tape, and ruler), Optiscan forms (Optiscan, Inc, Phoenix, Ariz) for gathering data, and passive consent forms were delivered to the schools. Each school had a designated representative responsible for conducting measures who was trained on the use of equipment. Most representatives were school nurses, but physical education teachers, health teachers, and school counselors also were used. Two or three days before data collection began, students in the selected classes were read a prepared paragraph containing information about the study. Each student was given a passive consent form to take home to parents or guardians; any student returning a signed form did not participate in the study. There was no consequence for nonparticipation nor was there a reward for participation.

The protocol for making measurements required that the weight scale be placed on a hard, smooth surface; carpeted areas were not used. The scale was calibrated to zero before use and recalibrated after every 10th student. All students were weighed and measured in a location where the information gathered would be confidential. Other students were not able to read the scale or height measurement or hear a weight or height given. Height and weight rounded to the nearest whole inch or pound were recorded on the Optiscan form, along with age, sex, date of birth, racial or ethnic background, and the school code number. No allowance was made for weight of clothing; however, students were asked to remove belts, heavy jewelry, jackets, and shoes. No student names were written on the data collection forms.

### YRBSS 

The YRBSS is a biennial survey used by many states for monitoring six categories of health risk behaviors among adolescents: unintentional injuries and violence, tobacco use, alcohol and other drug use, sexual behavior, physical activity, and diet and weight control (including questions about height and weight). It is a self-administered questionnaire survey, conducted under the supervision of a class teacher. The YRBSS was conducted from February through March 2003 by the Mississippi State Department of Health. The CAYPOS was designed to follow the same procedures established by CDC for the YRBSS.

The sampling frame for the YRBSS consisted of all Mississippi public middle schools including grades 6 through 8 (N = 729 schools) using fall 2002 enrollment numbers by grade. As with CAYPOS, the sample was selected using PC Sample software (28), which selected schools by probability proportional to enrollment in grades 6 through 8 with a random start. The selected sample consisted of 45 schools. Within selected schools, all classes in a required subject or all classes meeting during a particular period of the day (depending on the school) were listed. Classes were then selected by systematic equal probability sampling with a random start. Student participation was voluntary (using passive consent, as in CAYPOS), anonymous, and confidential.

### Data analysis 

Both the YRBSS and the CAYPOS used body mass index (BMI) criteria for defining overweight in children and youth that are slightly different from criteria used among adults. A more conservative definition of overweight is used because body fatness changes with age and differs between girls and boys. Guidelines used in this study were those recommended by CDC (29). Children or adolescents were considered overweight if their BMI was the 95th percentile or higher by age and sex and at risk for becoming overweight if their BMI was the 85th percentile or higher but less than the 95th percentile by age and sex.

For boys and girls separately, BMI-for-age *z* scores and percentiles were calculated using a public-domain SAS program based on the 2000 CDC growth charts (30). Point estimates of prevalence were calculated using SAS version 8.2 (SAS Institute, Inc, Cary, NC) and rounded to one decimal place. Ninety-five percent confidence intervals were calculated using SAS-callable SUDAAN version 8.0.1 (Research Triangle Institute, Research Triangle Park, NC), which takes into account the complex sample survey methodology in calculating variance estimates. Results were weighted to account for student and school nonresponse and adjusted by poststratification weighting so that results were representative of all public elementary and middle school students in the state. For the poststratification weighting in CAYPOS, single imputation of values for sex, race, and grade was used for 48 students with missing data for one or more of these variables. Prevalence estimates were compared by chi-square tests of association and trend.

## Results

### CAYPOS 

Thirty-seven (65%) of the 57 randomly selected schools agreed to participate in the CAYPOS. The overall response rate was 62% (school response rate [65%] x student response rate [96%]). After 29 students without data on date of birth or weight were excluded, the final data set used in the analysis consisted of 1658 students, including 855 boys (52%) and 777 girls (48%) (data missing for 26 students) and 773 whites (47%) and 862 nonwhites (53%) (data missing for 23 students). Table 1 shows the sex and race of students in the sample. Because 95% of nonwhite students were African American, the category *nonwhite* can be considered synonymous with African American in this report. The number of individuals in other race categories was too small for a separate analysis.

Twenty-four percent of students in grades 1 through 8 were found to be overweight, and another 14.7% were at risk for becoming overweight, giving a combined total of 38.7%, or nearly four out of 10 students at or above the 85th percentile (Table 2). Boys had a higher prevalence (26.4%) of overweight than girls (21.5%) (*P* = .07), and nonwhite students had a higher prevalence (26.2%) of overweight than white students (21.9%) (*P* = .11). White and nonwhite boys had very similar rates of overweight, whereas the prevalence of overweight among nonwhite girls was more than nine percentage points higher than that among white girls. The prevalence estimates for at risk for becoming overweight followed a different pattern. Nonwhite girls had the highest rate (18.6%) of at risk for becoming overweight, but the rate was not statistically different from the rate for white girls (*P* = .43). Nonwhite boys had the lowest prevalence of at risk for becoming overweight (9.2%).

In first grade, 17.5% of students were overweight, whereas 31.3% of students in eighth grade were overweight (Table 3). The prevalence of overweight increased steadily from grade 1 through grade 5; the prevalence was much lower in grade 6 but increased again through grade 8. The overall trend was not statistically significant (*P* = .17).

### Comparison of CAYPOS and YRBSS results

Thirty-four (76%) of 45 randomly selected middle schools participated in the 2003 Mississippi YRBSS (conducted in February 2003). The overall response rate was 65% (school response rate [76%] x student response rate [86%]); 1510 students were selected and participated. After excluding 302 students without data on height or weight, the final data set used in the analysis consisted of 1208 middle school students.

**Figure 1:**
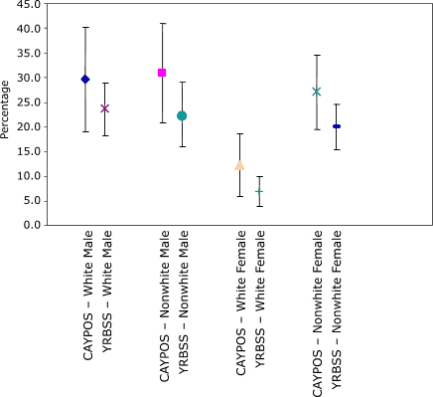
Comparison of 95% confidence intervals for prevalence of overweight among students in grades 6 through 8 from the Child and Youth Prevalence of Overweight Survey (CAYPOS) and the Youth Risk Behavior Surveillance System (YRBSS), by sex and race, Mississippi, 2003.

**Figure 2:**
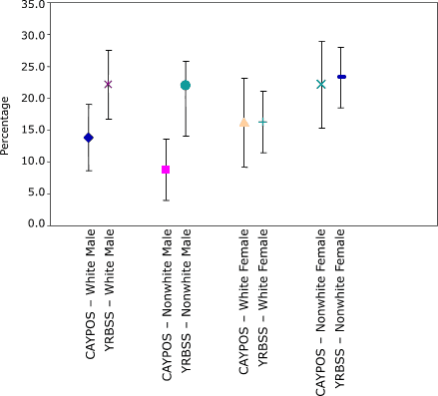
Comparison of 95% confidence intervals for prevalence of at risk for becoming overweight among students in grades 6 through 8 from the Child and Youth Prevalence of Overweight Survey (CAYPOS) and the Youth Risk Behavior Surveillance System (YRBSS), by sex and race, Mississippi, 2003.

The CAYPOS sample included 483 students from 17 middle schools (Table 1). In CAYPOS, 25.2% of students in grades 6 through 8 were found to be overweight, and another 15.2% were at risk of overweight, compared with 18.5% overweight and 20.7% at risk of overweight in the YRBSS. In all subgroups (with the exception of sixth-grade students), estimates of the prevalence of overweight from CAYPOS were higher than estimates from the YRBSS. However, in all cases, the 95% confidence intervals were wide and overlapped (Figure 1 and Table 4). Estimates of the prevalence of at risk for becoming overweight from the CAYPOS were lower than estimates from the YRBSS. Again, in most cases, the confidence intervals overlapped (Figure 2 and Table 4). The difference in the point estimates between the two surveys was large (approximately 13 percentage points): 31.3% from CAYPOS compared with 17.9% from YRBSS in grade 8 and 27.7% from CAYPOS compared with 14.7% from YRBSS in grade 7.

**Table:** 

**Group**	**CAYPOS, % (95% CI)**	**YRBSS, % (95% CI)**
White male	29.7 (19.1-40.3)	23.6 (18.2-29.0)
Nonwhite male	31.0 (20.8-41.1)	22.6 (16.1-29.2)
White female	12.2 (5.8-18.6)	6.9 (3.8-10.0)
Nonwhite female	27.1 (19.5-34.7)	20.0 (15.4-24.5)

**Table:** 

**Group**	**CAYPOS, % (95% CI)**	**YRBSS, % (95% CI)**
White male	13.8 (8.5-19.1)	22.2 (16.9-27.6)
Nonwhite male	8.8 (4.0-13.6)	22.0 (14.2-25.9)
White female	16.2 (9.2-23.1)	16.3 (12.0-20.6)
Nonwhite female	22.2 (15.4-28.9)	23.3 (18.5-28.1)

## Discussion

Data from CAYPOS suggest that the prevalence of overweight and at risk for becoming overweight among children and youth in grades 1 through 5 are higher than previously suspected, and in grades 6 through 8, overweight was higher than previously reported. A major finding is that a high percentage of children (one in four) are already overweight or at risk for becoming overweight in first grade, and the percentage of overweight increases through grade 5. Students experience a slight drop in BMI during sixth grade; however, this age is approximately when puberty begins, and many are experiencing a growth spurt. In grades 7 and 8, the combined rate is again higher than 40%. African American girls had the highest combined rate of overweight and at risk for becoming overweight (44.9%), with white boys having the highest rate of overweight (26.7%).

These findings are strikingly similar to those recently reported in Arkansas (25), where researchers found that the combined prevalence of overweight and at risk for becoming overweight peaked in grades 5 through 7 at 42%, with the highest rates among Hispanic (46%) and African American (41%) students. The combined prevalence for students in grades 1 through 8 in Arkansas ranged from 34% to 42%, compared with 39% among students in grades 1 through 8 in Mississippi.

Likewise, Hoelscher et al (26) collected height and weight data on students in grades 4, 8, and 11 in Texas and found the highest overweight rates among Hispanic and African American students and those in fourth grade. Twenty-two percent of students in grade 4 were overweight compared with 25.8% of students in the same grade in Mississippi. Similarly, in a study of elementary students in New York City, Thorpe et al (27) found the highest overweight rates among Hispanic boys and girls and African American girls, with 24% of students in grade 4 being overweight.

Unlike data from the YRBSS, data compared among these studies were based on actual height and weight measures rather than self-report. When compared with the YRBSS for middle school students, the CAYPOS overall rate of overweight was higher (25.2% rate from CAYPOS vs 18.5% rate from YRBSS) but the overall at-risk rate was lower (15.2% from CAYPOS vs 20.7% from YRBSS). The combined rates were similar, however (40.4% from CAYPOS vs 39.2% from YRBSS), suggesting that heavier students may not accurately self-report their height and weight, placing themselves in the at-risk category rather than in the overweight category.   

This is the first study to determine rates of overweight and at risk for becoming overweight using measured height and weight data from grades 1 through 8 and to compare these data with existing YRBSS data on middle school students. Although previous research has found self-reported height, weight, and BMI calculated from these values to be highly reliable over time, the BMIs based on self-reported height and weight values among the same sample were discrepant from measured height, weight, and BMIs calculated from measured values (31). One limitation of the current study was that actual discrepancies between the findings of the two separate studies (YRBSS and CAYPOS) were not established. 

Another limitation of this study is that it only included public school students, and thus the findings should not be generalized to all children and youth in these age groups. Given the similarity of the findings to other anthropometric surveys and the large differences between measured and self-reported data, we believe that future surveys should use anthropometric procedures in all grades, from kindergarten through 12th. This study was able to collect the data with limited funding, requiring only the purchase of equipment to measure the heights and weights, limited travel to provide training on data collection, and staff to enter and analyze the data. Therefore, consideration should be given to collecting data on all children, rather than just a sample, as is being done in Arkansas (25).

One of the *Healthy People 2010* (32) goals is to reduce the percentage of overweight children and youth to 5% from the 11% of children and youth aged 6 to 19 who were overweight or obese in 1988 to 1994. From the data in this study, it is clear that the problem is more pervasive than previously thought. Consequently, isolated and compartmentalized programs in select grades are not likely to adequately address this problem.

Data in this study suggest that intervention may need to begin earlier than the age of school entry and needs to be more comprehensive. More accurate data by age, grade, sex, and race will enable school health education and promotion goals to be better prioritized and targeted to those at greatest risk. Once interventions are designed and implemented, these data will assist in the evaluation of the impact and effectiveness of those programs.

## Figures and Tables

**Table 1:** Race and Sex of Students in Sample, Child and Youth Prevalence of Overweight Survey (CAYPOS) and Youth Risk Behavior Surveillance System (YRBSS), Mississippi, 2003

**Study**	**Boys No. (%)**	**Girls No. (%)**	**Total No. (%)**
**CAYPOS (grades 1-8)^a^**			
White	407 (25.2)	362 (22.4)	769 (47.7)
Nonwhite^b^	437 (27.1)	407 (25.2)	844 (52.3)
Total	844 (52.3)	769 (47.7)	1613 (100)
**CAYPOS (grades 6-8)^c^**			
White	104 (21.9)	99 (20.9)	203 (42.8)
Nonwhite^b^	137 (28.9)	134 (28.3)	271 (57.2)
Total	241 (50.8)	233 (49.2)	474 (100)
**YRBSS (grades 6-8)**			
White	278 (23.0)	289 (23.9)	567 (46.9)
Nonwhite	320 (26.5)	314 (26.0)	634 (52.5)
Total	602 (49.8)	606 (50.2)	1208 (100)

a Data on sex, race, or both were missing for 45 of the 1658 students in grades 1-8.

b Ninety-five percent of nonwhite students were African American.

c Data on sex, race, or both were missing for 9 of the 483 students in grades 6-8.

**Table 2:** Percentage of Overweight and At Risk for Becoming Overweight, Child and Youth Prevalence of Overweight Survey (CAYPOS), Mississippi, 2003

**Characteristic**	**At Risk for Overweight^a^% (95% CI)**	***P* Value^b^**	**Overweight^c^% (95% CI)**	** *P* Value^b^**
**Sex**				
Male	12.2 (9.9-14.6)	.007	26.4 (22.5-30.4)	.07
Female	17.3 (14.8-19.7)		21.5 (19.0-24.0)	
**Race**				
White	15.5 (13.0-18.0)	.39	21.9 (18.3-25.5)	.11
Nonwhite^d^	13.8 (11.0-16.7)		26.2 (22.9-29.5)	
**Race and sex**				
White male	15.1 (11.3-18.9)	.03	26.7 (20.7-32.7)	.89
Nonwhite^d^ male	9.2 (6.4-12.1)		26.2 (20.9-31.4)	
White female	16.0 (12.0-20.0)	.43	16.9 (13.8-20.1)	.005
Nonwhite^d^ female	18.6 (14.7-22.4)		26.3 (22.0-30.5)	
**Total**	14.7 (12.8-16.6)	NA	24.0 (21.6-26.4)	NA

CI indicates confidence interval; NA, not applicable.

a Body mass index (BMI) ≥85th percentile and <95th percentile for age and sex.

b For chi-square test.

c BMI ≥95th percentile for age and sex.

d Ninety-five percent of nonwhite students were African American.

**Table 3:** Percentage of Overweight and At Risk for Becoming Overweight, by School Grade, Child and Youth Prevalence of Overweight Survey (CAYPOS), Mississippi, 2003

**Grade**	**At Risk for Becoming Overweight^a^% (95% CI)^b^**	**Overweight^c^% (95% CI)^b^**
1	9.4 (6.1-12.6)	17.5 (12.9-22.1)
2	10.5 (7.8-13.2)	21.2 (14.7-27.8)
3	18.6 (13.7-23.5)	24.2 (18.2-30.2)
4	17.1 (12.5-21.8)	25.4 (20.6-30.2)
5	16.4 (9.7-23.1)	28.3 (21.2-35.4)
6	17.7 (11.9-23.5)	16.9 (9.7-24.0)
7	15.7 (11.9-19.6)	27.7 (24.1-31.2)
8	11.9 (6.1-17.8)	31.3 (24.4-38.2)

CI indicates confidence interval.

a Body mass index (BMI) ≥85th percentile and <95th percentile for age and sex.

b Χ2^2^ test for trend: *P* = .09 for at risk of overweight, *P* = .17 for overweight.

c BMI ≥95th percentile for age and sex.

**Table 4:** Comparison of Percentage of Overweight and At Risk for Becoming Overweight Among Students from the Child and Youth Prevalence of Overweight Survey (CAYPOS) and the Youth Risk Behavior Surveillance System (YRBSS), by Sex, Race, and School Grade (6–8), Mississippi, 2003

**Characteristic**	** At Risk for Becoming Overweight^a^**		**Overweight^b^**		
	**CAYPOS% (95% CI)**	**YRBSS% (95% CI)**	**CAYPOS% (95% CI)**	**YRBSS% (95% CI)**	
**Sex**					
Male	11.2 (8.2-14.3)	21.1 (17.2-25.0)	30.4 (24.1-36.6)		22.9 (18.6-27.1)
Female	19.3 (15.5-23.0)	20.3 (17.3-23.2)	19.9 (14.7-25.1)		14.0 (10.9-17.2)
**Race**					
White	15.0 (10.9-19.1)	19.3 (16.1-22.6)	21.0 (12.5-29.5)		15.4 (12.2-18.7)
Nonwhite^c^	15.4 (10.2-20.5)	21.7 (18.7-24.6)	29.1 (22.9-35.2)		21.3 (17.0-25.6)
**Race and sex**					
White male	13.8 (8.5-19.1)	22.2 (16.9-27.6)	29.7 (19.1-40.3)		23.6 (18.2-29.0)
Nonwhite^c^ male	8.8 (4.0-13.6)	22.0 (14.2-25.9)	31.0 (20.8-41.1)		22.6 (16.1-29.2)
White female	16.2 (9.2-23.1)	16.3 (12.0-20.6)	12.2 (5.8-18.6)		6.9 (3.8-10.0)
Nonwhite^c^ female	22.2 (15.4-28.9)	23.3 (18.5-28.1)	27.1 (19.5-34.7)		20.0 (15.4-24.5)
**School grade**					
6	17.7 (11.9-23.5)	21.7 (15.2-28.2)	16.9 (9.7-24.0)		23.8 (16.1-31.5)
7	15.7 (11.9-19.6)	23.1 (18.4-27.8)	27.7 (24.1-31.2)		14.7 (11.6-17.9)
8	11.9 (6.1-17.8)	18.7 (14.9-22.4)	31.3 (24.4-38.2)		17.9 (14.4-21.4)
**Total**	15.2 (12.1-18.3)	20.7 (18.8-22.6)	25.2 (20.4-30.0)		18.5 (15.6-21.5)

CI indicates confidence interval.

a Body mass index (BMI) ≥85th percentile and <95th percentile for age and sex.

b BMI ≥95th percentile for age and sex.

c Ninety-five percent of nonwhite students were African American.
